# Tissue factor targeted near-infrared photoimmunotherapy: a versatile therapeutic approach for malignancies

**DOI:** 10.1007/s00262-024-03903-2

**Published:** 2025-01-03

**Authors:** Seiichiro Takao, Hiroshi Fukushima, Aki Furusawa, Takuya Kato, Shuhei Okuyama, Makoto Kano, Hiroshi Yamamoto, Motofumi Suzuki, Miyu Kano, Peter L. Choyke, Hisataka Kobayashi

**Affiliations:** Molecular Imaging Branch, Center for Cancer Research, National Cancer Institute, NIH, 10 Center Drive, Bethesda, MD 20892 USA

**Keywords:** Tissue factor, Factor III, Near-infrared photoimmunotherapy, Preclinical model

## Abstract

Tissue factor (TF) is a cell surface protein that plays a role in blood clotting but is also commonly expressed in many cancers. Recent research implicated TF in cancer proliferation, metastasis, angiogenesis, and immune escape. Therefore, TF can be considered a viable therapeutic target against cancer. Herein, we developed and tested a TF-targeted near-infrared photoimmunotherapy (NIR-PIT) as a potential treatment for several types of cancer. Tisotumab, a TF antibody, was conjugated to IR700. The efficacy of TF-targeted NIR-PIT was investigated using multiple cancer cell lines (A431; epidermoid carcinoma, HPAF-II; pancreatic adenocarcinoma, HSC-2; oral carcinoma, HT1376-luc; bladder carcinoma, MDAMB231; breast adenocarcinoma, and SKOV3-luc; ovarian serous cystadenocarcinoma) in vitro. In vivo, the efficacy of TF-targeted NIR-PIT was evaluated in HPAF-II and A431 xenograft mouse models. Pathologic changes in these tumors after NIR-PIT were evaluated in these tumor models. All cancer lines demonstrated TF expression in vitro and in vivo. Additionally, TF expression was documented to localize to cancer cells in tumors. In vitro, TF-targeted NIR-PIT caused cell death in a light dose-dependent manner. In vivo, TF-targeted NIR-PIT suppressed tumor growth and improved survival rates compared to controls. Furthermore, in vivo NIR-PIT showed histological signs of cancer cell damage, such as cytoplasmic vacuolation, nuclear dysmorphism, and extracellular leakage of LDHA consistent with cell death. In conclusion, TF-targeted NIR-PIT holds promise as a treatment for multiple cancer models expressing TF, spanning multiple cancer types.

**Supplementary Information:**

The online version contains supplementary material available at 10.1007/s00262-024-03903-2.

## Introduction

Despite major advances in the treatment of cancer including antibody drug conjugates (ADCs) and immune modulatory therapies among others, new targeted treatments are still needed to eradicate most cancers [1–5]. Tissue factor (TF), also known as Factor III, CD142, or thromboplastin encoded by F3 gene, is a transmembrane glycoprotein commonly expressed on the cell surface membrane [6]. TF is a cofactor for coagulation activity factor VII/FVIIa and plays a crucial role in the coagulation cascade [7–10]. In malignancy, TF expression is often upregulated downstream of KRAS mutations and p53 inactivation, two of the most prevalent genetic alterations in human cancers [11, 12]. TF expression is, therefore, a common feature of numerous cancers [13] and has been implicated in cancer proliferation, metastasis, angiogenesis, and immune escape [6, 10, 13–17]. Thus, TF is an attractive therapeutic target for cancer treatment.

Tisotsumab vedotin is an ADC that targets TF that received accelerated approval from the US Food and Drug Administration in September 2021 for the treatment of adult patients with recurrent or metastatic cervical cancer who has disease progression during or after chemotherapy [18]. Its role in other solid cancers is undergoing clinical trials (NCT03485209, NCT04697628). ADCs are attractive because they theoretically target cancer cells preferentially that overexpress the target [19]. However, ADCs are not without problems, preeminently resistance due to payload drug efflux transporters, poor internalization, and tumor heterogeneity as well as serious off-target effects due payload instability [20, 21]. Therefore, while the principle of antibody targeting remains sound, alternative antibody-based therapies are being sought.

Near-infrared photoimmunotherapy (NIR-PIT) is a cell-specific cancer treatment that relies first on the binding of antibody-photoabsorber conjugate (APC) to cellular membranes. Subsequent NIR light-induces a photochemical reaction that results in membrane destruction and immunogenic cell death [22, 23]. The monoclonal antibodies that are conjugated to the photoabsorber IRDye700DX (IR700), a silica-phthalocyanine dye, are used to target antigens expressed on the surface of cancer cells [23]. After intravenous infusion APCs bind to cancer cells within 24 h, once the tumor is irradiated with NIR light the APCs change from hydrophilic to hydrophobic as the axial ligands of IR700 dissociate [24]. This photochemical reaction leads to aggregation of APCs, resulting in significant damage to the cancer cell membrane, without damage to the surrounding normal tissues. The first clinical application of NIR-PIT uses an anti-epidermal growth factor receptor (EGFR) antibody conjugated to IR700 in patients with head and neck cancer. This treatment has been approved for clinical use in Japan and is currently being evaluated in a Phase III clinical trial in the USA (LUZERA-301, NCT03769506). However, besides EGFR, a wide range of antigens can be targeted, thus broadening the potential applications of NIR-PIT even to non-EGFR-expressing tumors. In this study, we investigated TF-targeted NIR-PIT and assessed its efficacy using multiple cancer models, to evaluated if TF can be a new target of NIR-PIT.

## Materials and methods

### Patient cohorts for tissue microarray (TMA) and transcriptome profiling

TMAs of human pancreatic cancer surgical specimens (serial no. PA242e and PA805c) were purchased from Biomax (Rockville, MD, USA). Overlapping cores from the same case and cores in which 4',6-diamino-2-phenyl indole (DAPI) staining failed were excluded from analysis. Altogether, cores of 68 pancreatic carcinoma patients were evaluated for which clinical staging, pathological staging, and histologic grade were known. Baseline demographics of patients are shown in Table 1.

**Table 1:** Baseline characteristics of pancreatic cancer cases in tissue microarray

		Pancreatic cancer (*n* = 68)
Age (years)	Median (Range)	56 (23–80)
Gender	Female/ Male	27 (40%)/41 (60%)
Clinical Stage	IA / IB / IIA / IIB / III / IV	2 (3%)/24 (35%)/28 (41%)/12 (18%)/0 (0%)/2 (3%)
T stage	T1 / T2 / T3	2 (3%)/29 (43%)/37 (54%)
N stage	N0 / N1	56 (82%)/12 (18%)
Tumor Grade	Low / High / invalid	38 (56%)/26 (38%)/4 (6%)

Transcriptome data publicly available on this cancer cohort were used for the analysis between TF mRNA expression and overall survival in this study. Kaplan–Meier curves were performed using available gene expression data from 33 cancer types (*n* = 11,506) and pancreatic cancer (*n* = 181) from The Cancer Genome Atlas (TCGA) Pan-Cancer (Pancan) and Pancreatic Cancer (PAAD) datasets of the TGCA Project obtained from the University of California Santa Cruz Xena Browser [25]. Overall survival and TF mRNA expression data were downloaded from each cohort, duplicate samples were removed, and data were analyzed. Clinical staging, pathological staging, and histologic grade data were also downloaded from PAAD cohort.

### Reagents

IRDye700DX NHS ester was obtained from LI-COR Bioscience (Lincoln, NE, USA). Tisotumab, an anti-human TF recombinant monoclonal antibody (mAb), was purchased from Creative Biolabs Inc. (Shirley, NY, USA).

### Synthesis of IR-700-conjugated anti-TF antibody

IR700 was conjugated with tisotumab (0.5 mg) as previously reported [26]. The resulting antibody-photoabsorber conjugate (APC) is abbreviated as tiso-IR700. Tiso-IR700 was analyzed by SDS-PAGE with a 4–20% gradient polyacrylamide gel (Life Technologies, Gaithersburg, MD, USA). Unconjugated antibody was used as a control. The APCs were also assessed with size exclusion chromatography (SEC) by the method previously described [26].

### Cell culture

The information for human cancer cell lines, including culture conditions and sources, are shown in Table 2. All culture media were supplemented with 10% fetal bovine serum (Thermo Fisher Scientific, Waltham, MA, USA) and 100 IU/mL penicillin and streptomycin (Thermo Fisher Scientific). All cells were cultured in a humidified incubator at 37 °C in an atmosphere of 95% air and 5% carbon dioxide and used within 30 passages.

**Table 2:** List of cell lines used in this research

Cell line	Cancer type	Culture	Inoculation	Source
A431	Epidermoid carcinoma	DMEM	2.0 × 10^6^	ATCC
H460	Lung large cell carcinoma	RPMI	3.0 × 10^6^	ATCC
HPAF-II	Pancreatic ductal adenocarcinoma	EMEM	3.0 × 10^6^	ATCC
HSC-2	Oral cavity squamous cell carcinoma	DMEM	5.0 × 10^6^	Dr. Gary S. Goldberg (Rowan University)
HT1376-luc	Bladder carcinoma	DMEM	6.0 × 10^6^	FenicsBIO (Halethorpe, MD, USA)
MDAMB231	Breast adenocarcinoma	RPMI	10.0 × 10^6^	The Division of Cancer Treatment and Diagnosis Tumor Repository (NCI Frederick)
SKOV3-luc	Ovarian serous cystadenocarcinoma	McCoy's	3.0 × 10^6^	Caliper LifeSciences (Hopkinton, MA, USA)

*DMEM*, Dulbecco's modified eagle medium (ATCC, Manassas, VA, USA); *EMEM*, Eagle's minimum essential medium (ATCC); *RPMI*, RPMI1640 (Thermo fisher scientific, Rockford, IL, USA); *McCoy's*, McCoy's 5A medium (Thermo fisher scientific)

### In vitro TF expression analysis

In vitro TF expression of A431, H460, HPAF-II, HSC-2, HT1376-luc, MDAMB231, and SKOV3-luc was assessed by flowcytometry as previously described [27]. Cells were stained with PE-labeled anti-TF Ab (clone NY2, BioLegend, San Diego, CA) or PE-labeled mouse IgG1κ (clone MOPC-21, BioLegend) as well as fixable viability dye (Thermo Fisher Scientific). Relative fluorescence intensity (RFI) was defined as the ratio of specific fluorescence (mean fluorescence of target cells incubated with the anti-TF Ab) over non-specific fluorescence (mean fluorescence of target cells incubated with the mouse IgG1κ).

### In vitro cell-specific binding analysis

To verify the in vitro binding of tiso-IR700 to A431 cells or HPAF-II, 2.0 × 10^5^ cells were collected in 100 μL of PBS and incubated with 1 μg of tiso-IR700 for 30 min at 4 °C. To validate the specific binding of tiso-IR700, a tenfold molar excess of unconjugated tisotumab was added 30 min before the incubation with tiso-IR700. Dead cells were excluded from the analysis based on the staining with fixable viability dye. The fluorescence of the cells was analyzed by FACSLyric (BD Biosciences, San Jose, CA, USA) and FlowJo software (FlowJo LLC, Ashland, OR, USA). In addition, to visualize the in vitro binding tiso-IR700 to A431 cells or HPAF-II, 5.0 × 10^3^ on glass-bottomed dishes and incubated for 24 h. Cells were incubated with 100 μl fresh culture medium containing 1 μg tiso-IR700 for 1 h at 37 °C and observed with a fluorescence microscope (IX81; Olympus America, Center Valley, PA, USA). To validate the specific binding of tiso-IR700, a tenfold molar excess of unconjugated tisotumab was added 30 min before the incubation with tiso-IR700. Transmitted light DIC images were obtained, and IR700 was detected using the filter set, which included a 608–668 nm excitation filter and a 672–712 nm bandpass emission filter. The cells were then exposed to NIR light (690 nm, 150 mW/cm^2^, 50 J/cm^2^) using an ML7710 laser system (Modulight, Tampere, Finland). The DIC images were acquired again 15 min after NIR light irradiation.

### In vitro fluorescence microscopy

A431, HPAF-II, HSC-2, HT1376-luc, MDAMB231, and SKOV3-luc cells were seeded at 5.0 × 10^3^ on glass-bottomed dishes and incubated for 24 h. Cells were incubated with 100 μl fresh culture medium containing 1 μg tiso-IR700 for 1 h at 37 °C and observed with a fluorescence microscope. Transmitted light DIC images were obtained, and IR700 was detected using the filter set, which included a 608–668 nm excitation filter and a 672–712 nm bandpass emission filter. The cells were then exposed to NIR light (690 nm, 150 mW/cm^2^, 50 J/cm^2^) using an ML7710 laser system. The DIC images were acquired again 15 min after NIR light irradiation.

### In vitro NIR-PIT

A431, H460, HPAF-II, HSC-2, HT1376-luc, MDAMB231, or SKOV3-luc cells were seeded onto 24-well plates at 2.0 × 10^5^ per well in quadruplicate in 500 μL medium and incubated for 24 h. Cells were incubated with 500 μL fresh culture medium containing 1 μg tiso-IR700 for 1 h at 37 °C. After washing with PBS, phenol-red-free medium was added. NIR light (690 nm, 150 mW/cm^2^) using an ML7710 laser system was applied. To assess cell viability after NIR-PIT, cell proliferation was evaluated by 3-(4,5-Dimethyl-2-thiazolyl)-2,5-diphenyl-2H-tetrazolium bromide (MTT) assay previously described [28]. Each absorbance was measured at 570 nm on a microplate reader (Synergy H1; BioTek, Winooski, VT, USA). For relative quantification, the value of absorbance in each group was normalized to the control group. Moreover, cell surface expression of calreticulin and heat shock protein-70 (HSP70) was evaluated immediately after NIR-PIT. Cells were stained with following PE-labeled antibodies: anti-calreticulin antibody (rabbit poly) and rabbit IgG were obtained from Bioss Antibodies (Woburn, MA, USA); anti-HSP70 antibody (clone REA349) and human IgG1 isotype control (clone REA293) were obtained from Miltenyi Biotec (Gaithersburg, MD, USA). The fluorescence of cells was measured using FACSLyric and FlowJo software.

### Animal models

Female homozygote athymic nude mice, six to eight weeks old, were purchased from Charles River Laboratories. All cells were inoculated subcutaneously into the right dorsum of nude mice. Inoculated cell numbers are shown in Table 2. Tumor volumes were evaluated three times per week using TumorImager2™ (Biopticon, Princeton, NJ, USA) and calculated by TumorManager software (Biopticon). The mice were euthanized with inhalation of carbon dioxide gas when the tumor volume reached 1500 mm^3^ or the diameter was more than 2 cm.

#### In vivo TF expression analysis

To evaluate expression of TF in tumors, six tumor bearing mice (harboring one of the following tumor types: A431, H460, HSC-2, HT1376-luc, MDAMB231, or SKOV3-luc) were euthanized when the established tumor volume reached approximately 150 mm^3^. Single-cell suspensions from each tumor sample were prepared by the method previously described [27]. The cells were stained with the following antibodies: anti-CD31 (clone 390), anti-CD45 (clone 30-F11), anti-TF (clone NY2), anti-PDPN (clone 8.1.1), and mouse IgG1κ (clone MOPC-21) were obtained from BioLegend. Cells were also stained with fixable viability dye, and dead cells were gated out from the analysis. The fluorescence of the cells was then analyzed with FACSLyric and FlowJo software. Cancer cells were categorized as CD45-/ CD31-/PDPN-.

#### In vivo fluorescence imaging

Tumor bearing mice were injected with tiso-IR700 (50 ug) via lateral tail vein. Serial dorsal fluorescence images were obtained with the 700 nm fluorescence channel of a Pearl Imager (LI-COR Bioscience, Lincoln, NE, USA). The images were analyzed with Pearl Cam Software (LI-COR Bioscience). Regions of interest were drawn on the tumor and the non-tumoral region of the contralateral side. Target-to-background ratio (TBR) was calculated as (Mean fluorescence intensity of the tumor)/(Mean fluorescence intensity of the non-tumoral region of the contralateral side).

#### In vivo NIR-PIT

For subcutaneously inoculated HPAF-II xenograft models, mice were classified into three groups as follows: (1) no treatment (Control), (2) intravenous injection of tiso-IR700 only (APC-IV), and (3) intravenous injection of tiso-IR700 followed by NIR light irradiation (NIR-PIT). For subcutaneously inoculated A431 and H460 xenograft models, mice were classified into two groups as follows: (1) no treatment (Control) and (2) intravenous injection of tiso-IR700 followed by NIR light irradiation (NIR-PIT). For subcutaneous xenograft models, random mice grouping was performed based on tumor volumes using Tumorimager and TumorManager software (Biopticon). Tiso-IR700 (50 µg) was injected from seven to nine days after tumor inoculation. NIR light (690 nm, 150 mW/cm^2^, 50 J/cm^2^) was applied to the skin overlying the tumor 24 h after tiso-IR700 injection. When administering NIR light, the remainder of the mouse’s body was shielded by aluminum foil with a hole created so as to irradiate only the target tumor. 700 nm fluorescence and white light images were obtained before and after NIR-PIT using a Pearl Imager.

#### Histological analysis

Mice assigned into the Control and NIR-PIT groups were euthanized 24 h after NIR light irradiation. To assess histological changes after NIR-PIT, the tumor was harvested, and formalin-fixed, paraffin-embedded (FFPE) sections were prepared and stained with hematoxylin and eosin (HE) staining.

#### Multiplex immunohistochemistry (IHC)

Sections of TMA and FFPE xenografts were used for multiplex IHC using Opal Automation IHC Kit (Akoya Bioscience, Menlo Park, CA, USA) and Bond RXm autostainer (Leica Biosystems) [27]. The following antibodies and DAPI were used: anti-human CD45 (clone EP322Y, Cell Signaling Technology), anti-lactate dehydrogenase A (LDHA; clone C4B5, Cell Signaling Technology), anti-pan-cytokeratin (pCK; rabbit poly, Bioss Antibodies), anti-digoxigenin (DIG; clone 9H27L19, Thermo Fisher Scientific), anti-human TF (clone EPR22548-232, Abcam), and anti-human EGFR (clone E236, Abcam). Stained slides were analyzed with Mantra Quantitative Pathology Workstation (Akoya Biosystems) and inForm Tissue Finder software (Akoya Biosystems). To assess TF and EGFR expression in cancer cells, inForm software was trained to detect tissue and cell phenotypes using machine-learning algorithms based on the following criteria: areas with pCK expression = tumor, pCK + CD45- cells = cancer cells, respectively. For the expression analysis, specific TF and EGFR localized in the cell membrane of cancer cells were evaluated. inForm software computed H-scores based on membrane TF and EGFR in cancer cells. The average H-score was calculated from three images for each TMA; then, specimens were classified into four groups: negative (H-score 0–14), weak (H-score 15–99), moderate (H-score 100–199), and strong (H-score 200–300), as previously described [26, 29].

#### Detection of DIG-labeled Ab by multiplex immunohistochemistry

Tisotumab or anti-human IgG1-Kappa (Abinvivo, Shang Hai, China) was labeled with DIG by incubating 0.5 mg Ab and 25 μg DIG-NHS-ester (Thermo Fisher Scientific) using a similar method to IR700 conjugation. The resulting DIG-labeled Abs were abbreviated as tiso-DIG and isotype-DIG, respectively. Tumor bearing mice were injected with tiso-DIG or isotype-DIG (50 μg) into the lateral tail vein. Tumors were harvested 24 h after injecting DIG-labeled Abs. The distribution of DIG-labeled Abs was analyzed in FFPE sections by multiplex IHC using anti-DIG Ab.

#### Statistical analysis

Patient data from two TCGA cohorts were divided into high and low TF mRNA expression groups based on median. Continuous data were compared between two groups using the Mann–Whitney U test. A one-way analysis of variance (ANOVA) followed by Tukey’s test was performed to compare continuous data among groups of more than two. Tumor volumes were compared using a repeated measures two-way ANOVA followed by Tukey’s or Sidak’s test. The percent survival was plotted using the Kaplan–Meier method, and the results were compared using the log-rank test. When compared among groups of more than two, Bonferroni correction was performed. GraphPad Prism (GraphPad Software, La Jolla, CA, USA) was used for statistical analysis. A statistically significant result was defined as *p* < 0.05.

## Results

### TF is highly expressed in pancreatic cancer specimens

**Fig. 1:**
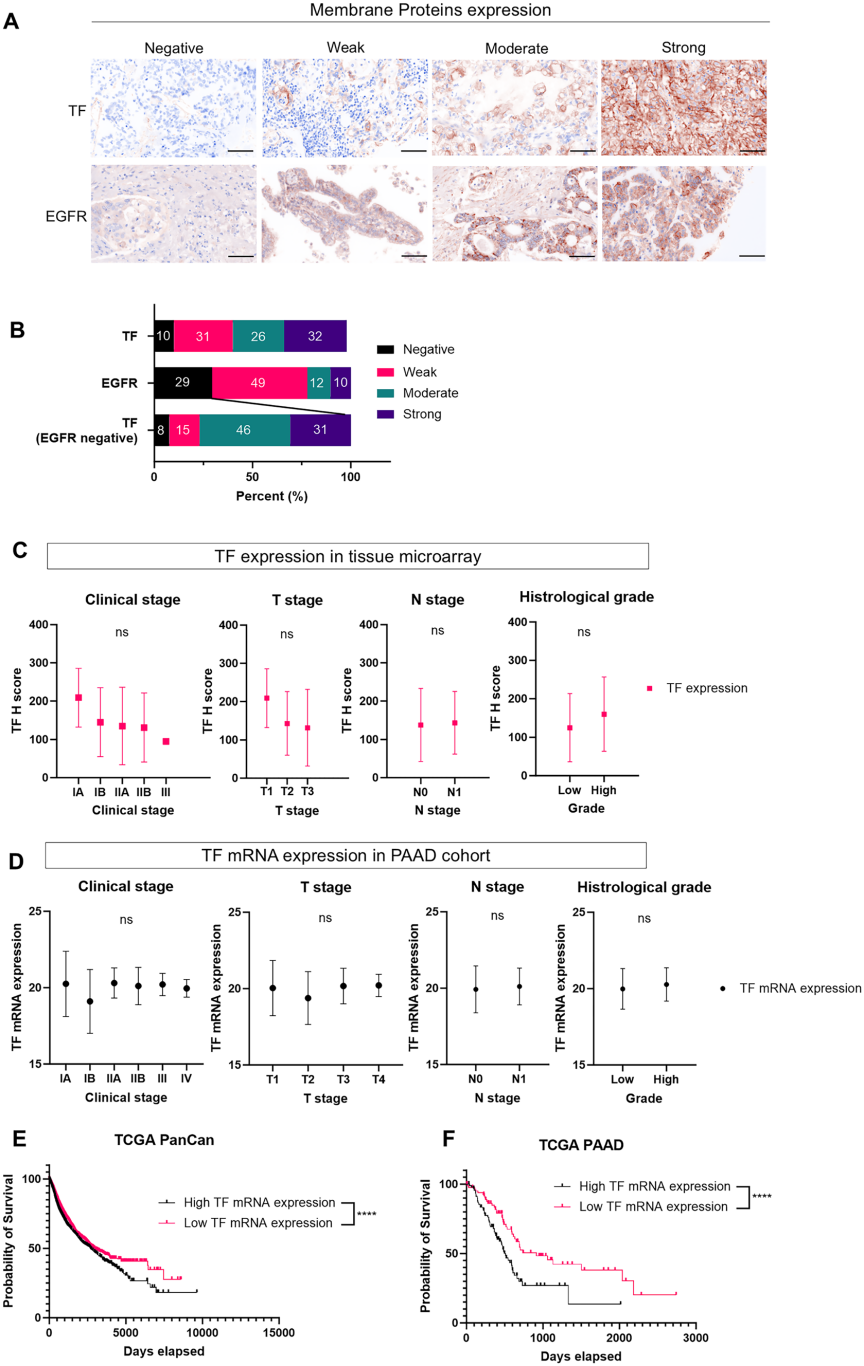
TF expression in tissue microarray and TCGA cohort. **A** and **B**, Immunohistochemical evaluation of membrane TF and EFGR expression in pancreatic cancer tissue microarray specimens. Both TF and EGFR H-scores were calculated based on membrane TF and EGFR expression. The staining was classified into one of four groups: negative (H-score 0–14), weak (H-score 15–99), moderate (H-score 100–199), and strong (H-score 200–300). **A**, Representative images are shown (images, × 200; scale bar, 100 μm). Antibody staining of TF or EGFR is shown in brown. Nuclei are stained with DAPI and shown in blue. **B**, Distribution of TF and EGFR staining category in 68 pancreatic cancer cases. **C** and **D**, Comparison of TF protein expression (H-score) and TF mRNA expression among the clinical stage, T stage, N stage, and tumor grading in tissue microarray [*n* = 68] or public clinical cohorts of pancreatic cancer [PAAD; TCGA 2019 (*n* = 182)]. G1 or G2 was defined as low-grade histology, and G3 or G4 was defined as high-grade histology. Mean and SD are shown as solid and dashed lines, respectively. ns, not significant (Clinical stage and T stage, one-way ANOVA followed by Tukey's test. N stage and histological grade, Mann Whitney U test). **E** and **F**, Kaplan–Meier overall survival curves of pan-cancer or pancreatic cancer patients according to TF mRNA expression in TCGA PanCan (*n* = 11,506) or TCGA PAAD dataset (*n* = 181). ****, *p* < 0.0001

We tested TF expression, along with EGFR expressions, in cancer tissue specimens. Pancreatic cancer was selected because it is one of the most representative refractory cancers. We investigated 68 pancreatic cancer TMA specimens for membrane TF and EGFR expression (Table 1) by IHC and calculated H-scores (Fig. 1A). TF and EGFR expression was categorized as negative, weak, moderate, or strong based on H-score. More specimens were categorized as strongly expressing TF than EGFR. Membrane TF expression was detected in 89% of overall pancreatic cancers and 92% of EGFR-negative pancreatic cancers (Fig. 1B).

In the same TMA, we also investigated the association between clinical stage and H-score, as well as the clinical information provided with the PAAD cohort and TF mRNA expression. There was no significant correlation between clinical staging, pathological staging, histological grade, and TF expression, neither in protein nor in mRNA (Fig. 1C and D). Furthermore, within the TCGA dataset (PanCan and PAAD), elevated TF mRNA expression in tumors was significantly associated with poor prognosis (Fig. 1E and F). We found that TF is commonly expressed in pancreatic cancer and its expression is an adverse prognostic factor.

### Cell surface TF expression in human cancer cell lines

**Fig. 2:**
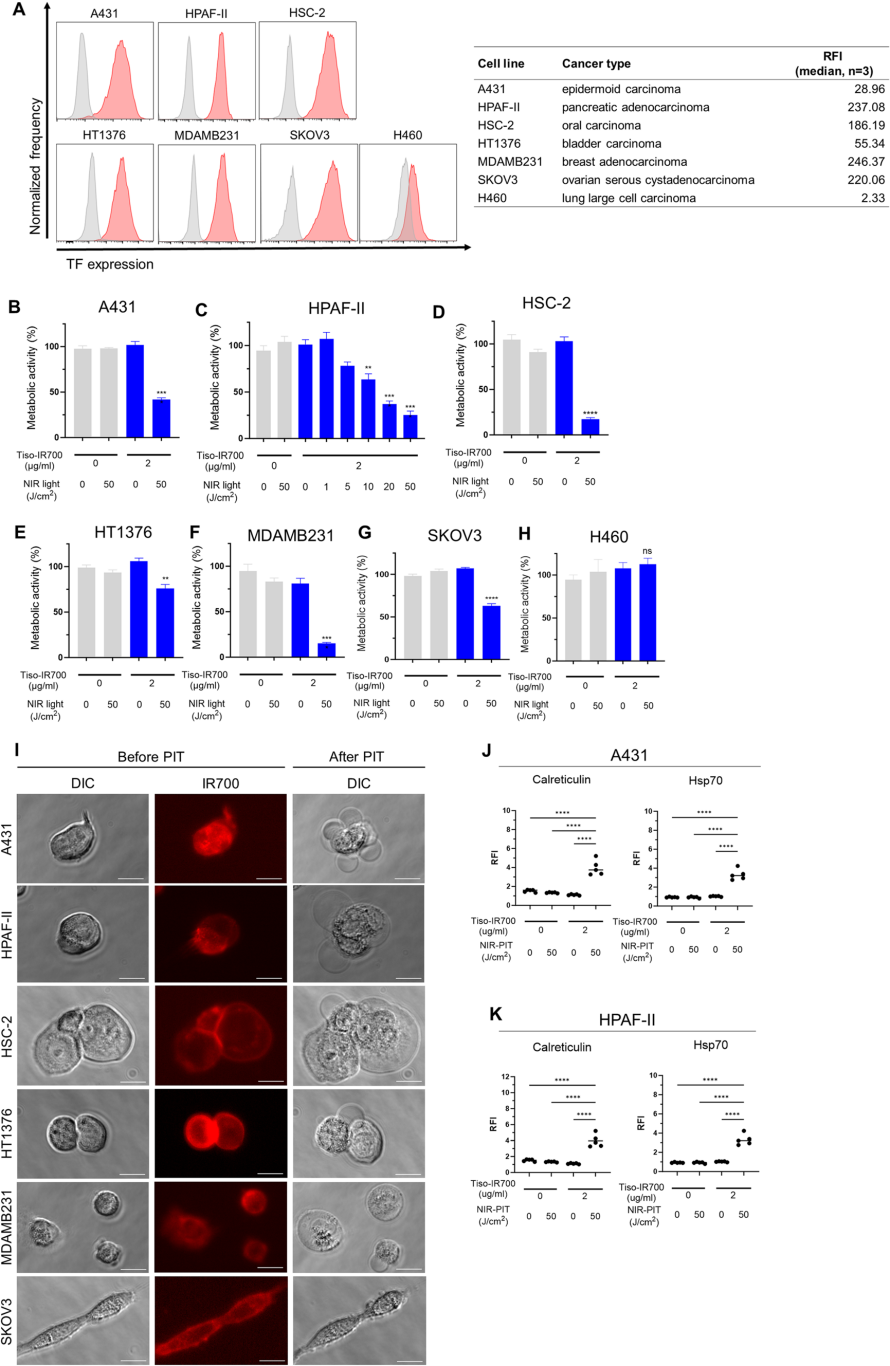
In vitro TF expression and efficacy of TF-targeted NIR-PIT in each cancer cell line. **A**, Flow cytometric analysis of in vitro TF expression on the cell surface of each cancer cell line. The relative fluorescence intensity (RFI) of TF for each cell line (*n* = 3; median) is shown in the right side table. RFI was calculated as the ratio of the median fluorescence intensity of anti-TF antibody to that of the isotype control. **B**–**H**, Metabolic activity of A431 (**B**), HPAF-II (**C**), HSC-2 (**D**), HT1376-luc (**E**), MDAMB231 (**F**), SKOV3-luc (**G**), and H460 (**H**) cells after in vitro TF-targeted NIR-PIT was measured by MTT assay (*n* = 4; one-way ANOVA followed by Tukey’s test). *, *p* < 0.05; **, *p* < 0.01; ***, *p* < 0.001; ****, *p* < 0.0001; ns, not significant vs. untreated control. **I**, Microscopic observation of cancer cells before and after in vitro NIR-PIT using tiso-IR700 (images, × 400; scale bar, 20 μm). DIC, differential interference contrast. **J** and **K**, Expression of calreticulin and Hsp70 after TF-targeted NIR-PIT in A431 (**J**) and HPAF-II (**K**). ****, *p* < 0.0001

Expression of TF on the surface of A431, H460, HPAF-II, HSC-2, HT1376-luc, and SKOV3-luc cells was evaluated in vitro. TF was highly expressed in A431, HPAF-II, HSC-2, HT1376-luc, MDAMB231, and SKOV3-luc cells, whereas low expression was detected in H460 cells (Fig. 2A).

### Specific binding of tiso-IR700 in A431 and HPAF-II

Conjugated tiso-IR700 was analyzed by SDS-PAGE and SEC. In SDS-PAGE, tiso-IR700 had the same approximate molecular weight as unconjugated tisotumab, but only tiso-IR700 exhibited 700-nm fluorescence (Fig. S1A). In SEC analysis, tiso-IR700 showed evidence of absorption at a wavelength of both 280 nm and 689 nm (Fig. S1B). These results verified successful conjugation of tiso-IR700.

Next, to assess the binding of tiso-IR700 to A431 and HPAF-II cells in vitro, these cells were incubated with tiso-IR700 and analyzed by flow cytometry. A431 and HPAF-II cells showed a high IR700 fluorescence signal (Fig. S2A). These signals were completely blocked by adding an excess of non-conjugated tisotumab (Fig. S2A), indicating that tiso-IR700 binds specifically to TF on the surface of A431 and HPAF-II cells. Microscopically, both A431 and HPAF-II cells incubated with an excess of non-conjugated tisotumab showed no tiso-IR700 binding cell surfaces, and both of them didn’t show morphologic changes in vitro TF-targeted NIR-PIT (Fig. S2B).

### Efficacy of in vitro TF-targeted NIR-PIT across various cancer cells

We examined the cytotoxicity of in vitro TF-targeted NIR-PIT across various cancer cells. In the MTT assay, cell viability was significantly decreased after TF-targeted NIR-PIT in all cell lines except H460, which had low TF expression (Fig. 2B–H), suggesting that TF-targeted NIR-PIT can kill various types of cancer cells as long as TF is expressed on the cell surface to some extent. Additionally, the cytotoxic efficacy of NIR-PIT increased in a light dose-dependent manner in HPAF-II cells (Fig. 2C). Microscopically, cells showed morphologic changes such as cellular swelling and bleb formation within 30 min following in vitro TF-targeted NIR-PIT (Fig. 2I). These findings are consistent with the known mechanism of action of NIR-PIT wherein cell membrane damage is caused by the NIR-triggered photochemical reaction. Cell surface calreticulin and HSP70 expression were significantly increased after NIR-PIT, suggesting that the cell death by TF-targeted NIR-PIT is immunogenic (Figs. 2J and K, and S3A–D).

### TF is expressed in established tumors in vivo

We tested TF expression in established tumors of A431, H460, HPAF-II, HSC-2, HT1376-luc, MDAMB231, and SKOV3-luc tumor model using flow cytometric staining. TF was expressed in all cancer cells within established tumors (Fig. S4A).

Immunohistochemistry analysis was performed in the established tumors for TF expression, and all the tumors showed TF expression (Fig. S4B). This result was consistent with flow cytometry results.

### Delivery of tisotumab and biodistribution of tiso-IR700 in vivo

**Fig. 3:**
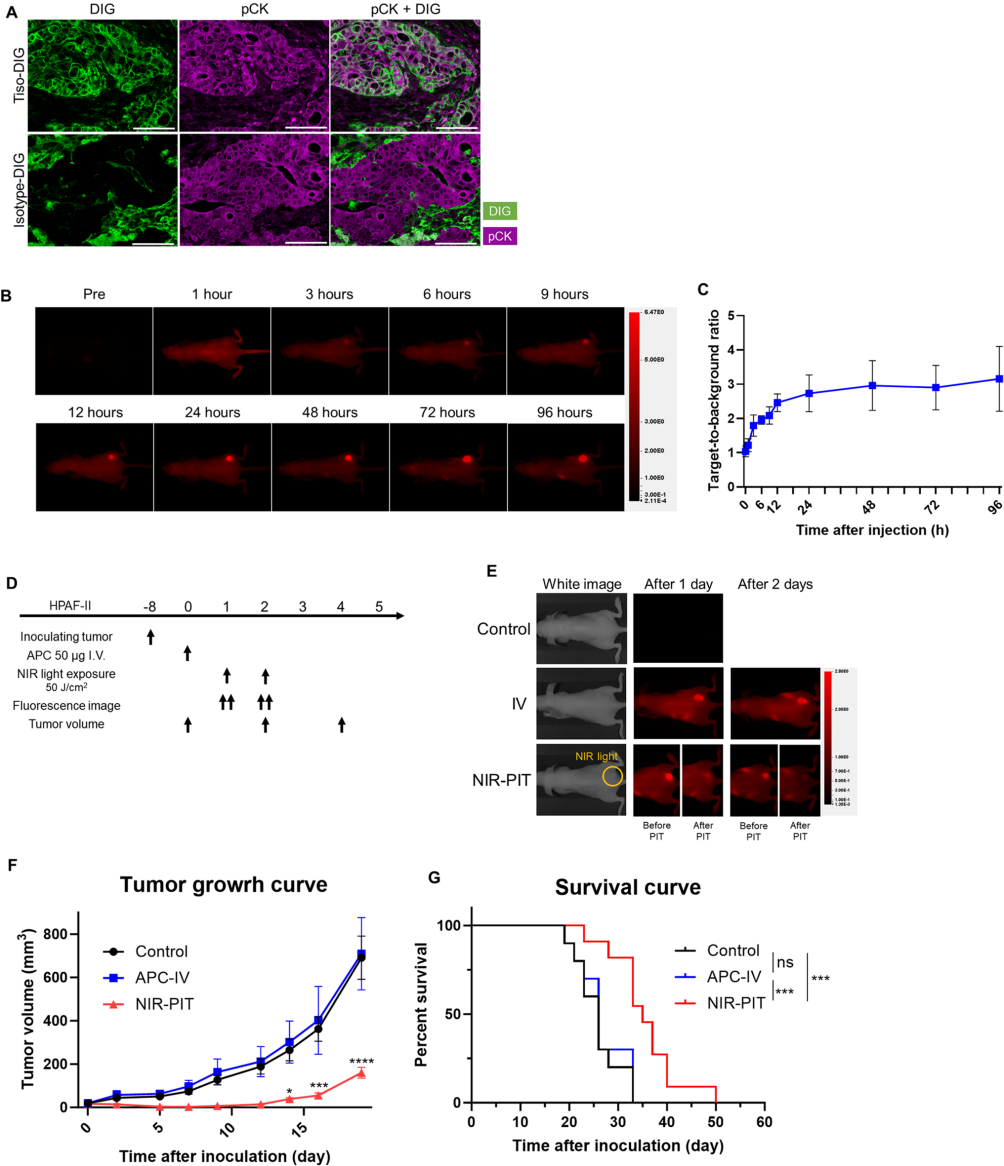
Delivery of tisotumab or tiso-IR700 and efficacy of in vivo TF-targeted NIR-PIT in the HPAF-II subcutaneous xenograft model. **A**, Tumors were harvested 24 h after injecting tiso-DIG or isotype-DIG into mice and the distribution in HPAF-II tumors was examined by multiplex immunohistochemistry (*n* = 4; images, × 200; scale bar, 100 μm). DIG and pCK are shown in green and purple, respectively. tiso-DIG, digoxigenin (DIG)-conjugated tisotumab. **B**, In vivo fluorescence imaging of tiso-IR700. Representative fluorescence images at 700 nm in an HPAF-II tumor bearing mouse. All images were acquired at the indicated time points after injecting tiso-IR700 into mice. AU, arbitrary units. **C**, Quantitative analysis of target-to-background ratio after injecting tiso-IR700 (*n* = 5). **D**, Treatment schedule. **E**, Representative 700-nm fluorescence images before and after NIR-PIT. **F**, Tumor volume curves (*n* = 10–11; mean ± SEM; repeated measures two-way repeated measures ANOVA followed by Tukey's test); *, *p* < 0.05; **, *p* < 0.01; ***p < 0.001; ****, *p* < 0.0001 versus the Control group. **G**, Survival curves (*n* = 10–11, log-rank test with Bonferroni correction); ***, *p* < 0.001

To evaluate the delivery of tisotumab to cancer cells in vivo, either tiso-DIG or isotype-DIG was infused into HPAF-II tumor bearing mice, then intratumoral digoxigenin (DIG) distribution was analyzed by multiplex IHC. In TF expressing tumors, tiso-DIG was detected on the cell surface throughout the tumor tissue (Fig. 3A). Isotype-DIG was not detected in the tumor tissue but was detected in some cells in the stroma, likely mediated by Fc receptor binding. These results indicated that tisotumab was successfully delivered to the tumor tissue and then bound to the surface of HPAF-II tumors.

Serial in vivo fluorescent imaging of tiso-IR700 was performed after its injection in HPAF-II tumor bearing mice (Fig. 3B and C). The TBR of tiso-IR700 increased up to 24-h post-injection and remained stable thereafter in HPAF-II models during the observation period. To achieve higher fluorescence intensity at the tumor site and the highest TBR, NIR light irradiation was administered 24 h after the injection of tiso-IR700 in vivo studies.

### In vivo efficacy of TF-targeted NIR-PIT

To evaluate the in vivo therapeutic efficacy of TF-targeted NIR-PIT, we employed the HPAF-II pancreatic cancer model (Fig. 3D), in which high TF expression was observed (Fig. S4). A 700-nm fluorescent signal was clearly detected at the tumor site prior to therapeutic NIR light exposure. This signal decayed immediately after therapeutic NIR light irradiation, suggesting photoconversion of IR700 due to ligand exchange causing loss of fluorescence. However, over time, the fluorescence signal reaccumulated and immediately declined after the second NIR light irradiation (Fig. 3E). In the NIR-PIT group, tumor growth was significantly suppressed compared to the control and APC-IV groups (Figs. 3F and S5A). The NIR-PIT group showed significantly longer survival compared to the control and APC-IV groups (Fig. 3G). Tumor growth and survival were not significantly different between the control and APC-IV groups.

**Fig. 4:**
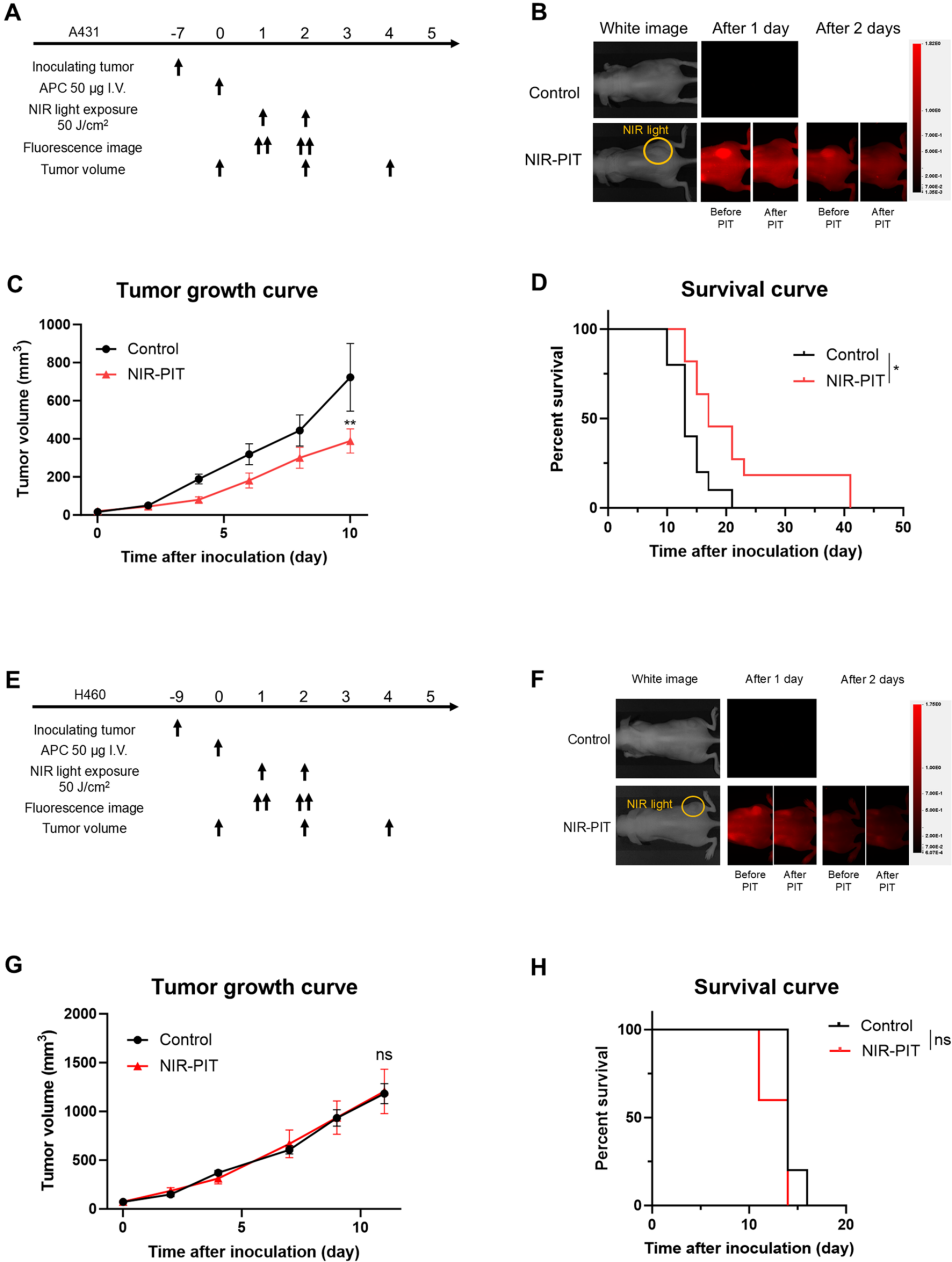
Efficacy of in vivo TF-targeted NIR-PIT in the A431 (**A-D**) and H460 (**E-H**) subcutaneous xenograft model. **A**, Treatment schedule of A431 model. **B**, Representative 700-nm fluorescence images before and after NIR-PIT. **C**, Tumor volume curves (*n* = 10; mean ± SEM; repeated measures two-way repeated measures ANOVA followed by Sidak's test); **, *p* < 0.01 versus the control group. **D**, Survival curves (*n* = 10, log-rank test); *, *p* < 0.05. **E**, Treatment schedule of H460 model. **F**, Representative 700-nm fluorescence images before and after NIR-PIT. **G**, Tumor volume curves (*n* = 10; mean ± SEM; repeated measures two-way repeated measures ANOVA followed by Sidak's test); ns, not significant versus the control group. **H**, Survival curves (*n* = 10, log-rank test); ns, not significant

Additionally, we analyzed the therapeutic efficacy of in vivo TF-targeted NIR-PIT in subcutaneously inoculated A431 (Fig. 4A) and H460 tumors (Fig. 4E). A431 exhibits high expression of TF whereas H460 exhibits low expression of TF (Fig. S4). In the A431 tumor xenograft model, a 700-nm fluorescent signal was clearly detected at the tumor site prior to therapeutic NIR light exposure. This fluorescence signal at the tumor site reaccumulated after NIR irradiation and immediately declined after the second NIR light irradiation, similar to HPAF-II cell models (Fig. 4B). NIR-PIT significantly suppressed tumor growth and improved survival compared to the control group (Figs. 4C, D and S5B), suggesting that it had a therapeutic effect similar to the HPAF-II NIR-PIT model.

In the H460 tumor xenograft model, which does not express TF, no reaccumulation of tiso-IR700 was observed (Fig. 4F), and no significant therapeutic efficacy was observed after NIR-PIT either in tumor growth or survival (Fig. 4G and 4H).

### Histological changes after in vivo NIR-PIT using tiso-IR700

**Fig. 5:**
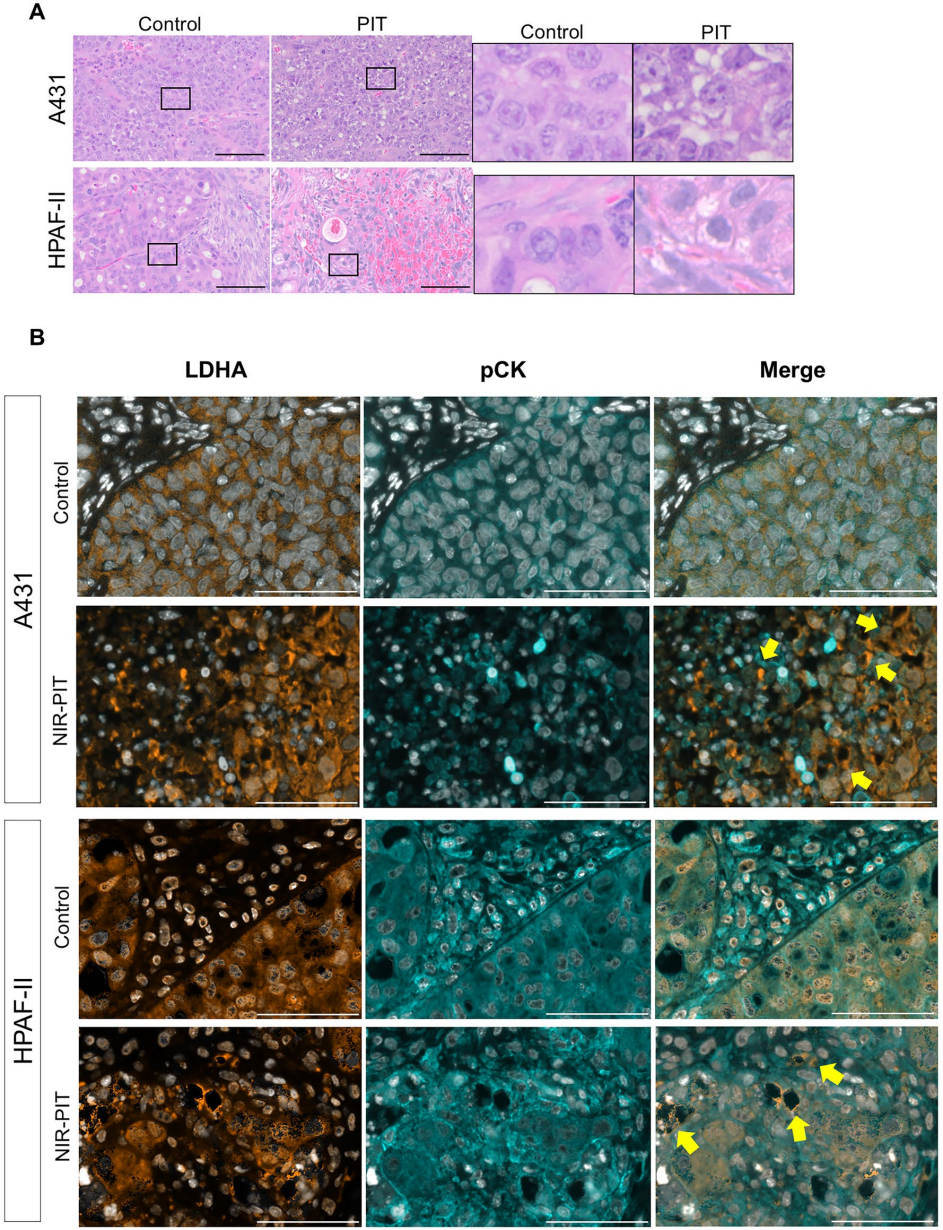
Histological changes after in vivo TF-targeted NIR-PIT. Tumor tissue histology was examined 24 h after NIR-PIT. **A**, H&E staining of A431 and HPAF-II tumors after NIR-PIT (images, × 200; scale bar, 100 μm). Insets are enlarged and displayed in the right panels. **B**, Immunohistochemical evaluation of lactate dehydrogenase A (LDHA) expression in A431 and HPAF-II tumors 24 h after NIR-PIT. Representative pictures of LDHA expression (images; × 200; scale bar, 100 μm). The inset shows examples of LDHA leakage into the extracellular space, which suggests necrotic cell death (yellow-filled arrow). Antibody staining of LDHA and pan-cytokeratin (pCK) is shown in orange and cyan, respectively. Nuclei are stained with DAPI and shown in white

We examined the histology of the tumors 24 h after NIR-PIT to evaluate the direct cytotoxic effects of in vivo NIR-PIT. On H&E staining, all tumors showed large numbers of necrotic cells which typically showed fewer darker nuclei than normal cells and eosinophilic cytoplasm with some vacuolar degeneration (Fig. 5A and S6A). Such histological changes were not seen in the control group. Furthermore, after NIR-PIT, multiplex IHC demonstrated the release of LDHA, which was localized in the cytoplasm of cancer cells in the control group (Figs. 5B and S6B), suggesting necrotic cell death [30].

## Discussion

We demonstrate that TF-targeted NIR-PIT has a marked anti-tumor effect across multiple malignancies, culminating in the induction of immunogenic cell death. This makes TF-targeted NIR-PIT a useful tool in the treatment of many different cancer types. Since TF expression was seen in many EGFR-negative tumors, TF-targeted NIR-PIT can be an alternative treatment choice for such cases.

IR700 fluorescent imaging showed that tiso-IR700 could circulate for at least four days, as shown in Fig. 3. Such prolonged circulation may have potential benefits and risks. As shown in Fig. 3E, the circulating tiso-IR700 can reaccumulate in the residual tumor after the initial NIR-PIT treatment. Although we tested up to two NIR light exposures in this study, the circulating probe potentially enables repeated treatments until complete ablation is achieved. In terms of the potential damage risk, the circulating probe can bind other normal tissues with TF expression. However, the damage to these tissues can be avoided by keeping patients from strong sunlight or any source of NIR light. Indeed, patients who receive EGFR-targeted NIR-PIT are recommended to stay away from sunlight for a week, allowing anti-EGFR-IR700 excretion. Additionally, IR700 is a water-soluble dye with no phototoxic or biotoxic properties; therefore, IR700 itself is safe and readily excreted in the urine, even if detached from tiso-IR700. Tisotumab also has an established safety profile, suggesting that circulating tiso-IR700 poses minimal risk for clinical NIR-PIT.

TF is broadly expressed across various types of cancer making it an ideal “general purpose” target. Moreover, circulating tumor cells (CTCs) also express TF and NIR-PIT may find a role in suppressing metastases by treating CTCs. TF-positive tumor cells commonly shed cells into the circulation which form CTCs through the activation of platelets and fibrin [17]. These CTCs are resistant to the cytotoxic effects of NK and T cells and also play a major role in tumor metastases and recurrences [17]. Because high numbers of CTCs are associated with a poor prognosis [31], the direct cytotoxic effect of TF-targeted NIR-PIT on cancer cells could suppress the production of CTCs from the tumor by efficiently killing TF-positive cells within the tumor. Therefore, it is possible that TF-targeted NIR-PIT, leading to immunogenic cell death in TF-positive cancer cells, has additional benefits for cancer patients.

Currently, EGFR-targeted NIR-PIT is the only clinically approved therapy of its kind. Although EGFR is expressed in many cancers, it does not comprehensively cover all refractory cancers. Previously, we demonstrated that a cocktail injection of two different antibody-IR700 conjugates created a more homogeneous microdistribution of antibody conjugates, resulting in enhanced therapeutic effects after NIR-PIT [32]. We suggest that tisotumab, which has already been approved for clinical use, might be an ideal additional agent to add, because both tisotumab and IR700 have safety profiles in the human body. Thus, TF-targeted NIR-PIT might be a stand-alone photoimmunotherapy agent or might be used in combination with EGFR-targeted NIR-PIT to make such treatments more effective.

There are several limitations to this study. First, since we used human cell line-derived xenografts in immunodeficient mice, the effect of NIR-PIT on the anti-cancer immune activation could not be demonstrated. We did, however, demonstrate immunogenic cell death after TF-targeted NIR-PIT. The study of the immune effects of TF NIR-PIT has to await the production of a suitable mAb against mouse TF that can be used in immunocompetent mouse tumor models. Previous studies have shown that anti-cancer immune responses were highly induced by NIR-PIT targeting EGFR [33], CD29 [34], and CD44 [35]. Thus, it is likely that TF-targeted NIR-PIT will have similar immune effects. Another issue is that we did not assess coagulation during NIR-PIT. Given that TF plays a crucial role in the coagulation cascade, there is a potential risk of bleeding when it is used as a therapy. In the present study, TF-targeted NIR-PIT only damaged cancer cells because tisotumab is a human anti-TF antibody and does not bind to mouse TF; thus, we could not assess if TF-targeted NIR-PIT affects coagulation. However, tisotumab has already been deployed in clinical settings and its safety for human use at doses higher than would be needed for NIR-PIT has shown no coagulation disorders among patients. Moreover, its paratope binds to a site unrelated to the coagulation system [36]. Given these factors, it is believed that the risk of bleeding associated with treatment with TF-targeted NIR-PIT is minimal. In addition, TF is typically not expressed in immune cells, except in a subset of monocytes [37]. This implies that TF-targeted NIR-PIT would not negatively affect the host’s anti-tumor immune response following immunogenic cell death after NIR-PIT.

In conclusion, TF-targeted NIR-PIT showed significant efficacy in vitro across multiple human cancer cell lines. Moreover, TF-targeted NIR-PIT demonstrated significant in vivo tumor control in murine xenograft models of pancreatic cancer and epithelioid cancer, as well as immunogenic cell death after treatment across multiple tumors. Therefore, TF-targeted NIR-PIT is a promising therapy for numerous malignancies and is a good candidate for clinical translation.

## Supplementary Information

Below is the link to the electronic supplementary material.

Supplementary file1 (DOCX 7596 KB)

## Data Availability

The data generated in this study are available within the article and its Supplementary Data or from the corresponding author upon reasonable request.

## References

[1] Berger MF, Mardis ER (2018) The emerging clinical relevance of genomics in cancer medicine. Nat Rev Clin Oncol 15:353–365. 10.1038/s41571-018-0002-6 29599476 10.1038/s41571-018-0002-6 PMC6658089

[2] Wei Q, Li P, Yang T et al (2024) The promise and challenges of combination therapies with antibody-drug conjugates in solid tumors. J Hematol Oncol 17:1. 10.1186/s13045-023-01509-2 38178200 10.1186/s13045-023-01509-2 PMC10768262

[3] Topalian SL, Drake CG, Pardoll DM (2015) Immune checkpoint blockade: a common denominator approach to cancer therapy. Cancer Cell 27:450–461. 10.1016/j.ccell.2015.03.001 25858804 10.1016/j.ccell.2015.03.001 PMC4400238

[4] Luo J (2021) KRAS mutation in pancreatic cancer. Semin Oncol 48:10–18. 10.1053/j.seminoncol.2021.02.003 33676749 10.1053/j.seminoncol.2021.02.003 PMC8380752

[5] Halbrook CJ, Lyssiotis CA, Pasca di Magliano M, Maitra A (2023) Pancreatic cancer: advances and challenges. Cell 186:1729–1754. 10.1016/j.cell.2023.02.014 37059070 10.1016/j.cell.2023.02.014 PMC10182830

[6] Han X, Guo B, Li Y, Zhu B (2014) Tissue factor in tumor microenvironment: a systematic review. J Hematol Oncol 7:54. 10.1186/s13045-014-0054-8 25084809 10.1186/s13045-014-0054-8 PMC4237870

[7] Dorgalaleh A, Bahraini M, Shams M et al (2023) Molecular basis of rare congenital bleeding disorders. Blood Rev 59:101029. 10.1016/j.blre.2022.101029 36369145 10.1016/j.blre.2022.101029

[8] Eisenreich A, Celebi O, Goldin-Lang P, Schultheiss HP, Rauch U (2008) Upregulation of tissue factor expression and thrombogenic activity in human aortic smooth muscle cells by irradiation, rapamycin and paclitaxel. Int Immunopharmacol 8:307–311. 10.1016/j.intimp.2007.06.005 18182245 10.1016/j.intimp.2007.06.005

[9] Eisenreich A, Rauch U (2010) Regulation and differential role of the tissue factor isoforms in cardiovascular biology. Trends Cardiovasc Med 20:199–203. 10.1016/j.tcm.2011.08.001 22137642 10.1016/j.tcm.2011.08.001

[10] Ahmadi SE, Shabannezhad A, Kahrizi A et al (2023) Tissue factor (coagulation factor III): a potential double-edge molecule to be targeted and re-targeted toward cancer. Biomarker Res 11:60. 10.1186/s40364-023-00504-6 10.1186/s40364-023-00504-6 PMC10242999 37280670

[11] Hassan N, Efing J, Kiesel L, Bendas G, Götte M (2023) The tissue factor pathway in cancer: overview and role of heparan sulfate proteoglycans. Cancers (Basel) 15(5):524. 10.3390/cancers15051524 36900315 10.3390/cancers15051524 PMC10001432

[12] Yu JL, May L, Lhotak V, Shahrzad S, Shirasawa S, Weitz JI, Coomber BL, Mackman N, Rak JW (2005) Oncogenic events regulate tissue factor expression in colorectal cancer cells: implications for tumor progression and angiogenesis. Blood 105:1734–1741. 10.1182/blood-2004-05-2042 15494427 10.1182/blood-2004-05-2042

[13] de Bono JS, Harris JR, Burm SM et al (2023) Systematic study of tissue factor expression in solid tumors. Cancer Rep (Hoboken) 6:e1699. 10.1002/cnr2.1699 36806722 10.1002/cnr2.1699 PMC9940005

[14] Breij EC, de Goeij BE, Verploegen S et al (2014) An antibody-drug conjugate that targets tissue factor exhibits potent therapeutic activity against a broad range of solid tumors. Cancer Res 74:1214–1226. 10.1158/0008-5472.Can-13-2440 24371232 10.1158/0008-5472.CAN-13-2440

[15] Hisada Y, Mackman N (2019) Tissue factor and cancer: regulation, tumor growth, and metastasis. Semin Thromb Hemost 45:385–395. 10.1055/s-0039-1687894 31096306 10.1055/s-0039-1687894 PMC6546519

[16] Hu Z (2018) Therapeutic antibody-like immunoconjugates against tissue factor with the potential to treat angiogenesis-dependent as well as macrophage-associated human diseases. Antibodies (Basel) 7(1):8. 10.3390/antib7010008 31105982 10.3390/antib7010008 PMC6519474

[17] Li H, Yu Y, Gao L, Zheng P, Liu X, Chen H (2022) Tissue factor: a neglected role in cancer biology. J Thromb Thrombolysis 54:97–108. 10.1007/s11239-022-02662-0 35763169 10.1007/s11239-022-02662-0

[18] Bogani G, Coleman RL, Vergote I, Raspagliesi F, Lorusso D, Monk BJ (2023) Tisotumab vedotin in recurrent or metastatic cervical cancer. Curr Probl Cancer 47:100952. 10.1016/j.currproblcancer.2023.100952 36842202 10.1016/j.currproblcancer.2023.100952

[19] Chau CH, Steeg PS, Figg WD (2019) Antibody-drug conjugates for cancer. Lancet 394:793–804. 10.1016/s0140-6736(19)31774-x 31478503 10.1016/S0140-6736(19)31774-X

[20] Abelman RO, Wu B, Spring LM, Ellisen LW, Bardia A (2023) Mechanisms of resistance to antibody–drug conjugates. Cancers 15(4):1278. 10.3390/cancers15041278 36831621 10.3390/cancers15041278 PMC9954407

[21] Khoury R, Saleh K, Khalife N, Saleh M, Chahine C, Ibrahim R, Lecesne A (2023) Mechanisms of resistance to antibody-drug conjugates. Int J Mol Sci 24(11):9674. 10.3390/ijms24119674 37298631 10.3390/ijms24119674 PMC10253543

[22] Mitsunaga M, Ogawa M, Kosaka N, Rosenblum LT, Choyke PL, Kobayashi H (2011) Cancer cell–selective in vivo near infrared photoimmunotherapy targeting specific membrane molecules. Nat Med 17:1685–1691. 10.1038/nm.2554 22057348 10.1038/nm.2554 PMC3233641

[23] Kobayashi H, Choyke PL (2019) Near-infrared photoimmunotherapy of cancer. Acc Chem Res 52:2332–2339. 10.1021/acs.accounts.9b00273 31335117 10.1021/acs.accounts.9b00273 PMC6704485

[24] Sato K, Ando K, Okuyama S et al (2018) Photoinduced ligand release from a silicon phthalocyanine dye conjugated with monoclonal antibodies: a mechanism of cancer cell cytotoxicity after near-infrared photoimmunotherapy. ACS Cent Sci 4:1559–1569. 10.1021/acscentsci.8b00565 30555909 10.1021/acscentsci.8b00565 PMC6276043

[25] Goldman MJ, Craft B, Hastie M et al (2020) Visualizing and interpreting cancer genomics data via the Xena platform. Nat Biotechnol 38:675–678. 10.1038/s41587-020-0546-8 32444850 10.1038/s41587-020-0546-8 PMC7386072

[26] Fukushima H, Takao S, Furusawa A et al (2024) Near-infrared photoimmunotherapy targeting Nectin-4 in a preclinical model of bladder cancer. Cancer Lett 585:216606. 10.1016/j.canlet.2023.216606 38272345 10.1016/j.canlet.2023.216606 PMC10923129

[27] Takao S, Fukushima H, King AP et al (2023) Near-infrared photoimmunotherapy in the models of hepatocellular carcinomas using cetuximab-IR700. Cancer Sci 114:4654–4663. 10.1111/cas.15965 37817415 10.1111/cas.15965 PMC10727998

[28] Fukushima H, Kato T, Furusawa A et al (2022) Intercellular adhesion molecule-1-targeted near-infrared photoimmunotherapy of triple-negative breast cancer. Cancer Sci. 10.1111/cas.15466 35723065 10.1111/cas.15466 PMC9459244

[29] Hoffman-Censits JH, Lombardo KA, Parimi V et al (2021) Expression of Nectin-4 in bladder urothelial carcinoma, in morphologic variants, and nonurothelial histotypes. Appl Immunohistochem Mol Morphol 29:619–625. 10.1097/pai.0000000000000938 33901032 10.1097/PAI.0000000000000938 PMC8429050

[30] Chan FK, Moriwaki K, De Rosa MJ (2013) Detection of necrosis by release of lactate dehydrogenase activity. Methods Mol Biol 979:65–70. 10.1007/978-1-62703-290-2_7 23397389 10.1007/978-1-62703-290-2_7 PMC3763497

[31] Baghban R, Roshangar L, Jahanban-Esfahlan R, Seidi K, Ebrahimi-Kalan A, Jaymand M, Kolahian S, Javaheri T, Zare P (2020) Tumor microenvironment complexity and therapeutic implications at a glance. Cell Commun Signal 18:59. 10.1186/s12964-020-0530-4 32264958 10.1186/s12964-020-0530-4 PMC7140346

[32] Nakajima T, Sano K, Choyke PL, Kobayashi H (2013) Improving the efficacy of photoimmunotherapy (PIT) using a cocktail of antibody conjugates in a multiple antigen tumor model. Theranostics 3:357–365. 10.7150/thno.5908 23781283 10.7150/thno.5908 PMC3677407

[33] Okada R, Kato T, Furusawa A, Inagaki F, Wakiyama H, Choyke PL, Kobayashi H (2021) Local depletion of immune checkpoint ligand CTLA4 expressing cells in tumor beds enhances antitumor host immunity. Adv Ther 4(5):2000269. 10.1002/adtp.202000269 10.1002/adtp.202000269 PMC8115697 33997271

[34] Furusawa A, Okada R, Inagaki F et al (2022) CD29 targeted near-infrared photoimmunotherapy (NIR-PIT) in the treatment of a pigmented melanoma model. Oncoimmunology 11:2019922. 10.1080/2162402x.2021.2019922 35003897 10.1080/2162402X.2021.2019922 PMC8741294

[35] Fukushima H, Furusawa A, Kato T, Wakiyama H, Takao S, Okuyama S, Choyke PL, Kobayashi H (2023) Intratumoral IL15 improves efficacy of near-infrared photoimmunotherapy. Mol Cancer Ther 22:1215–1227. 10.1158/1535-7163.Mct-23-0210 37461129 10.1158/1535-7163.MCT-23-0210 PMC10592297

[36] Theunissen JW, Cai AG, Bhatti MM, Cooper AB, Avery AD, Dorfman R, Guelman S, Levashova Z, Migone TS (2018) Treating tissue factor-positive cancers with antibody-drug conjugates that do not affect blood clotting. Mol Cancer Ther 17:2412–2426. 10.1158/1535-7163.Mct-18-0471 30126944 10.1158/1535-7163.MCT-18-0471

[37] Cimmino G, Ciccarelli G, Golino P (2015) Role of tissue factor in the coagulation network. Semin Thromb Hemost 41:708–717. 10.1055/s-0035-1564045 26408920 10.1055/s-0035-1564045

