# Supplementary material for: Tissue factor targeted near-infrared photoimmunotherapy: a versatile therapeutic approach for malignancies
Source: Cancer Immunol Immunother. 2025 Jan 3;74(2):48. doi: 10.1007/s00262-024-03903-2 (PMC11699179; doi:10.1007/s00262-024-03903-2)
Supplement: Supplementary file 1 — Supplementary file1 (DOCX 7596 KB) [file 262_2024_3903_MOESM1_ESM.docx]

Supplementary Data for

**Tissue Factor targeted Near-infrared Photoimmunotherapy: A versatile therapeutic approach for malignancies**

**Authors:** Seiichiro Takao^1^, Hiroshi Fukushima^1^, Aki Furusawa^1^, Takuya Kato^1^, Shuhei Okuyama^1^, Makoto Kano^1^, Hiroshi Yamamoto^1^, Motofumi Suzuki^1^, Miyu Kano^1^, Peter L. Choyke^1^, Hisataka Kobayashi^1^

**Affiliations:**

^1^Molecular Imaging Branch, Center for Cancer Research, National Cancer Institute, NIH, Bethesda, MD, 20892, USA

**Corresponding author:** Hisataka Kobayashi, M.D., Ph.D.

Molecular Imaging Branch, Center for Cancer Research, National Cancer Institute, NIH, 10 Center Drive, Bethesda, MD, 20892, USA

Tel: 240-858-3069; Fax: 240-541-4527; E-mail: [kobayash@mail.nih.gov](mailto:kobayash@mail.nih.gov)

**List of Supplementary Data**

Supplementary Fig. S1. Quality check of tiso-IR700.

Supplementary Fig. S2. Detection of tiso-IR700 bound to A431 and HPAF-II cells by flow cytometric and fluorescence microscope analysis.

Supplementary Fig. S3. Expression of calreticulin and Hsp70 after TF-targeted NIR-PIT in HSC-2, HT1376-luc, MDAMB231, and SKOV3-luc.

Supplementary Fig. S4. TF expression in tumors established from A431, HPAF-II, HSC-2, HT1376-luc, MDAMB231, and SKOV3-luc cell lines.

Supplementary Fig. S5. Representative images of tumors. In vivo TF-targeted NIR-PIT against HAPF-II and A431 tumor models.

Supplementary Fig. S6. Histological changes after in vivo TF-targeted NIR-PIT.


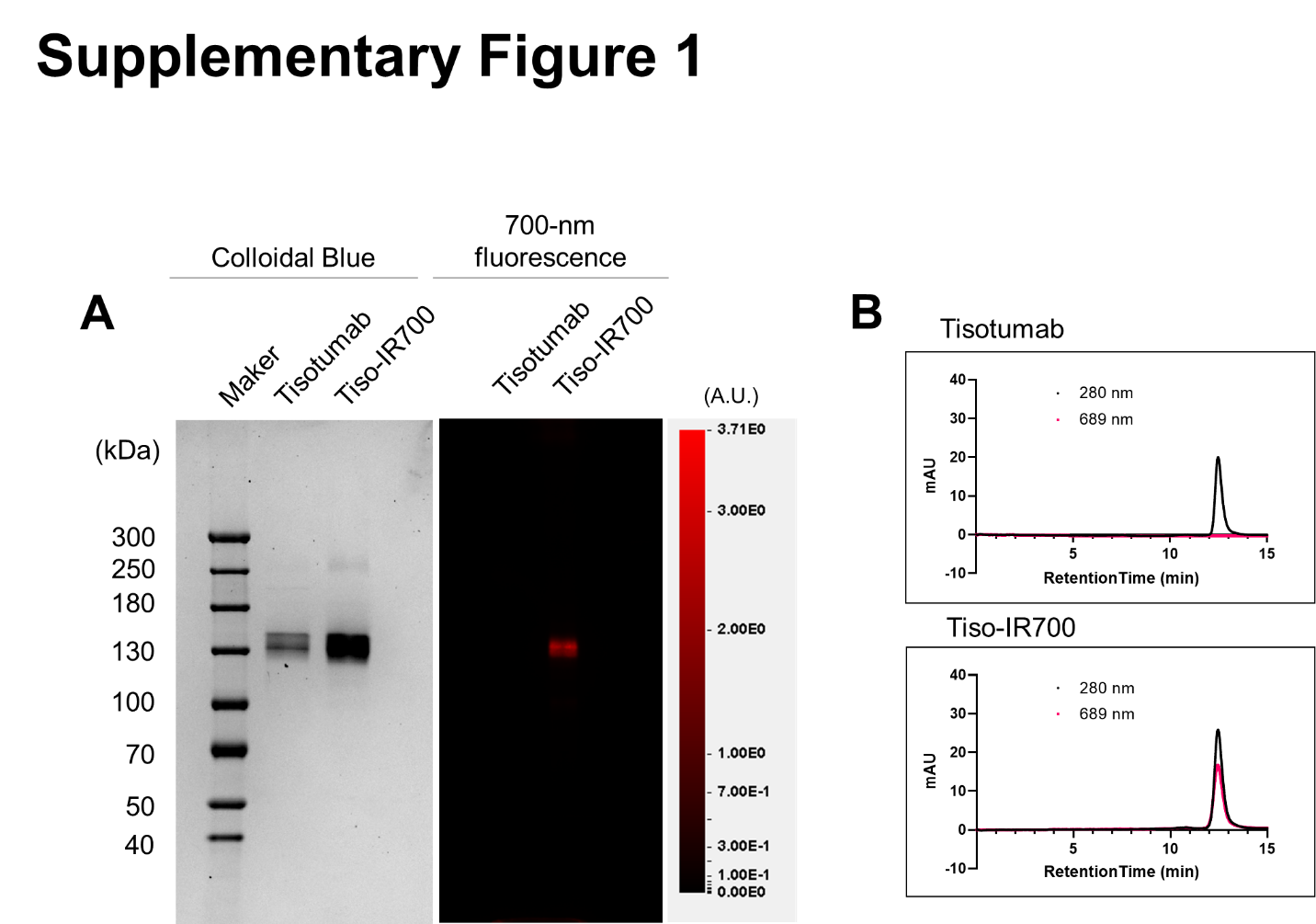


**Supplementary Fig. S1. Quality check of tiso-IR700.**

(A) SDS-PAGE (left, colloidal blue staining; right, 700 nm fluorescence). Unconjugated tisotumab was used as a control. Fluorescence intensity was confirmed in the band of tiso-IR700. Tiso-IR700 has the same approximate molecular weight as unconjugated tisotumab but only tiso-IR700 exhibited 700-nm fluorescence. A.U., arbitrary units. (B) Size exclusion chromatography of tiso-IR700. Tiso-IR700 showed evidence of absorption at a wavelength of both 280 nm and 689 nm.

**
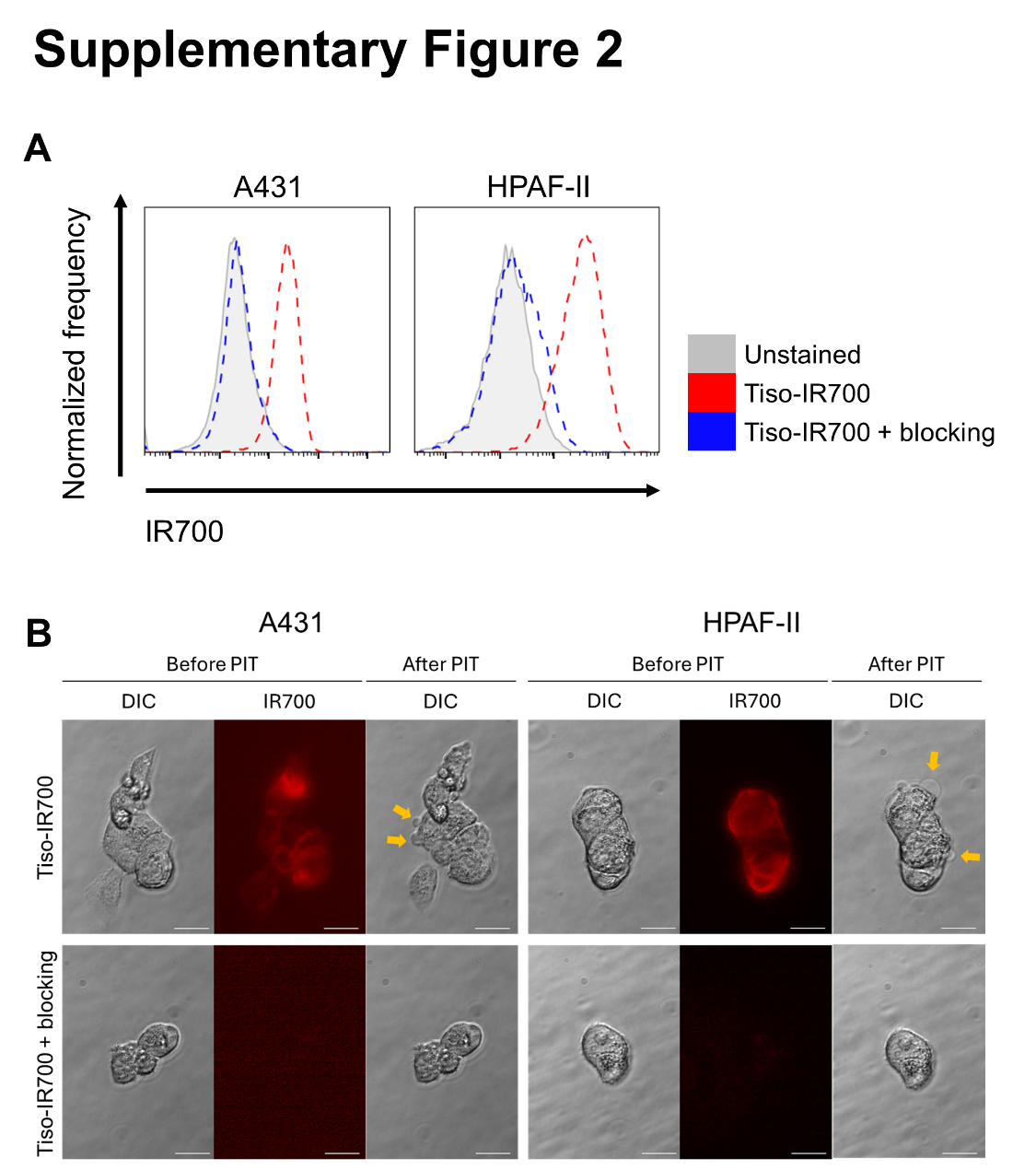
**

**Supplementary Fig. S2. Detection of tiso-IR700 bound to A431 and HPAF-II cells by flow cytometric analysis (A) and fluorescence microscopy (B).**

B, Microscopic observation of cancer cells before and after in vitro NIR-PIT using tiso-IR700 (images, ×400; scale bar, 20 μm). Cells showed morphologic changes such as cellular swelling and bleb formation (yellow-filled arrow). DIC, differential interference contrast.


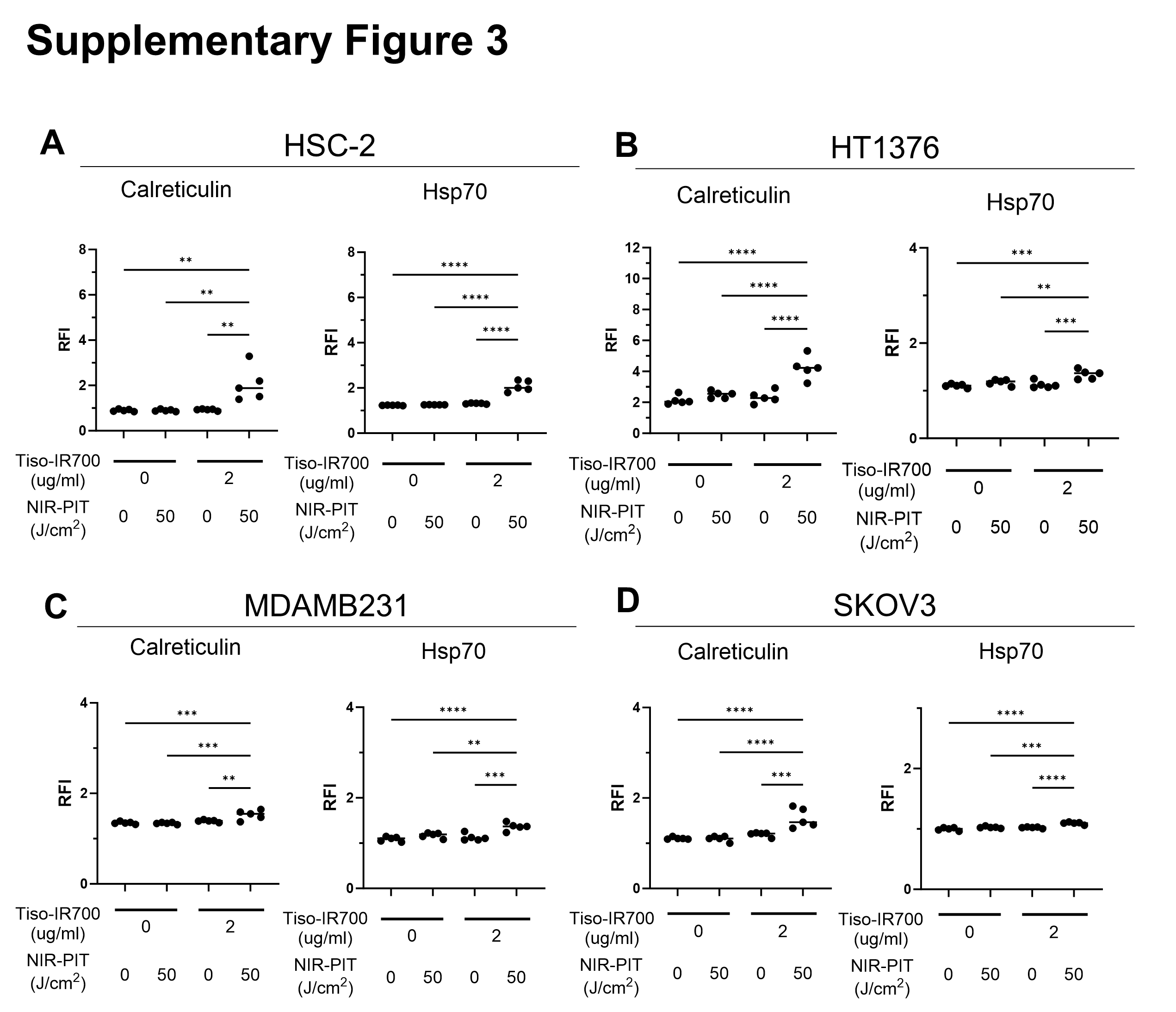


**Supplementary Fig. S3.** **Expression of calreticulin and Hsp70 after TF-targeted NIR-PIT in HSC-2 (A), HT1376-luc (B), MDAMB231 (C), and SKOV3-luc (D).**

Surface expression levels of calreticulin and HSP70 were determined by flow cytometry calculated as the RFI. (n = 5; one-way ANOVA followed by Tukey’s test); **, p < 0.01; ***, p< 0.001; ****, p<0.0001.


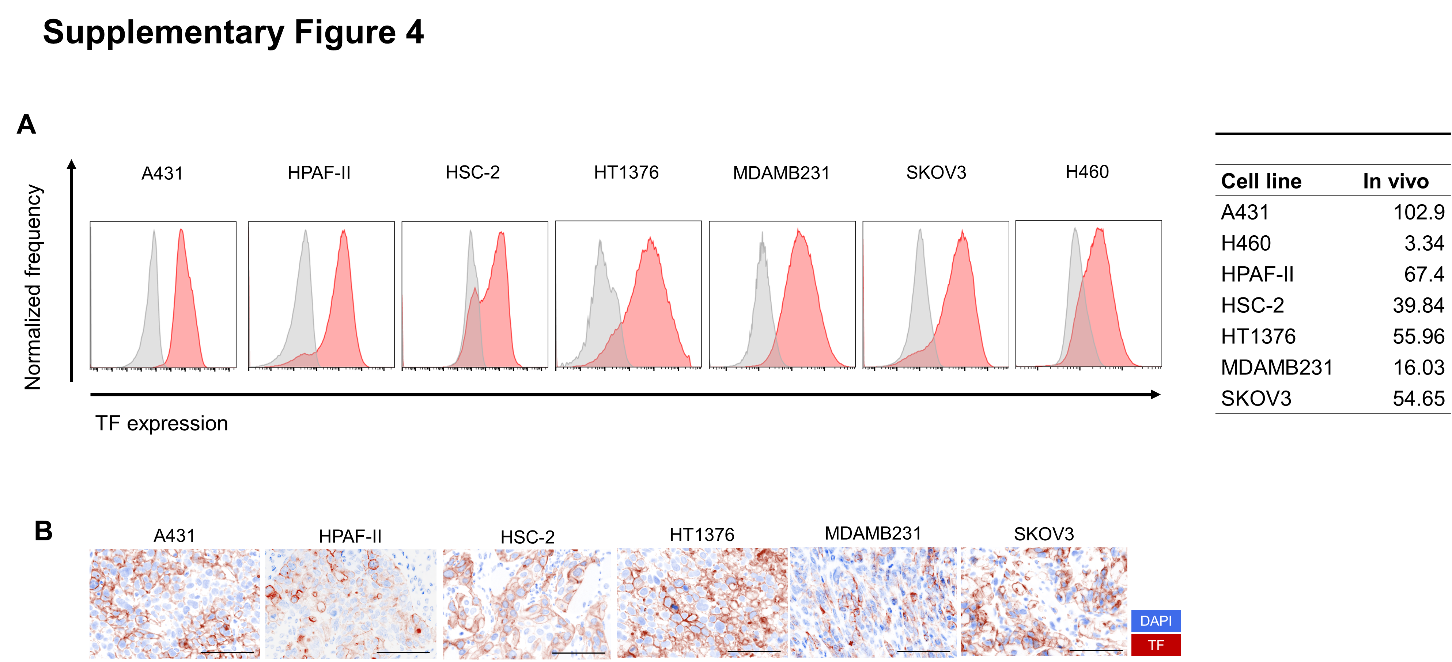


**Supplementary Fig. S4. In vivo TF expression in tumors established from A431, HPAF-II, HSC-2, HT1376-luc, MDAMB231, and SKOV3-luc cell lines.**

(A) Flow-cytometric analysis of *in vivo* TF expression on the cell surface of each cancer cell line. The relative fluorescence intensity (RFI) of TF for each cell line (n = 3; median) is shown right side table. RFI was calculated as the ratio of the median fluorescence intensity of anti-TF antibody to that of the isotype control. (B) Immunohistochemical evaluation of TF expression in A431, HPAF-II, HSC-2, HT1376-luc, MDAMB231, and SKOV3-luc tumors (images; ×200; scale bar, 100 μm). Antibody staining of TF is shown in brown, respectively. Nuclei are stained with DAPI and shown in blue.


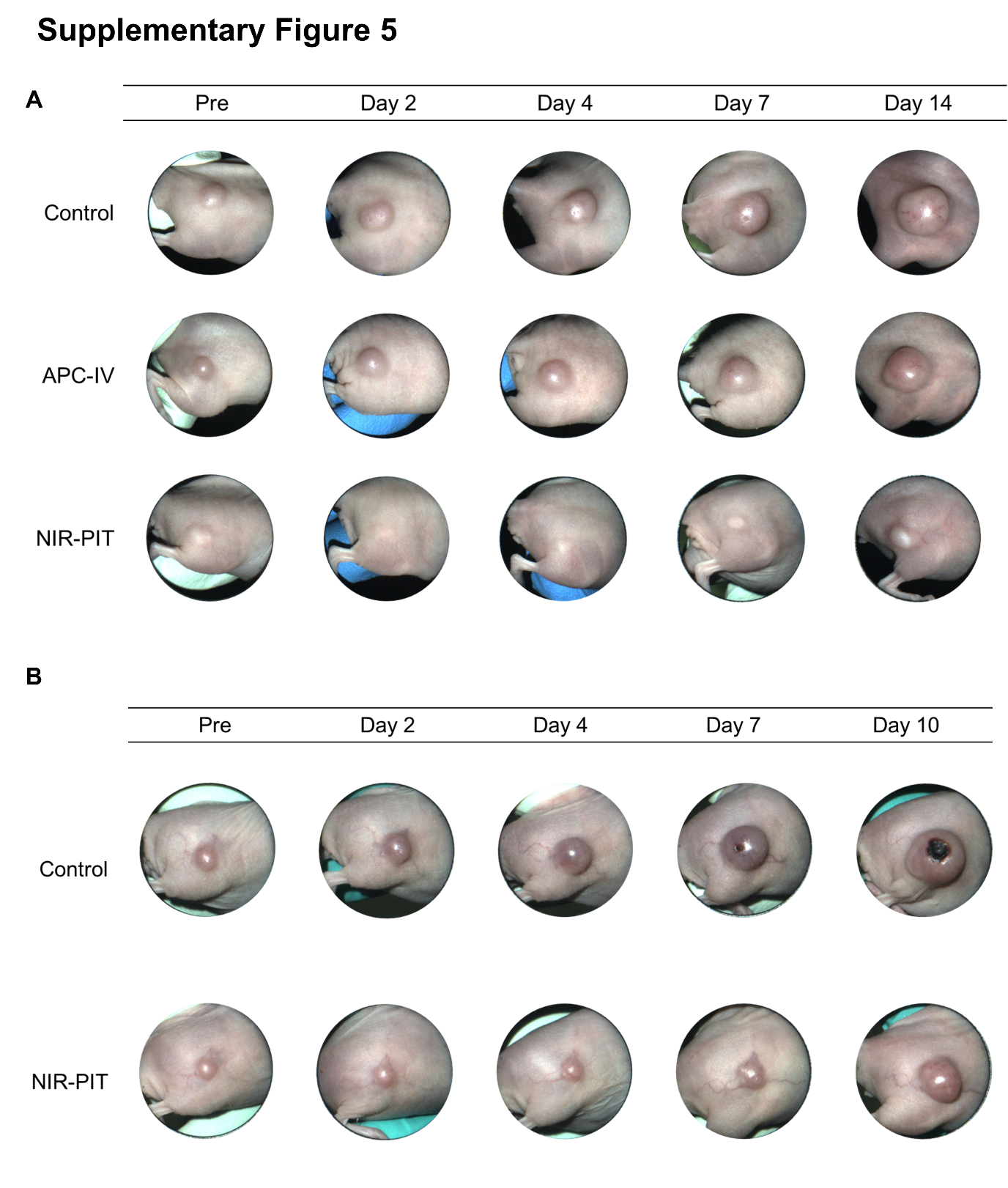


**Supplementary Fig. S5. Representative images of tumors. In vivo TF-targeted NIR-PIT against HAPF-II (A) and A431 (B) tumor models.**


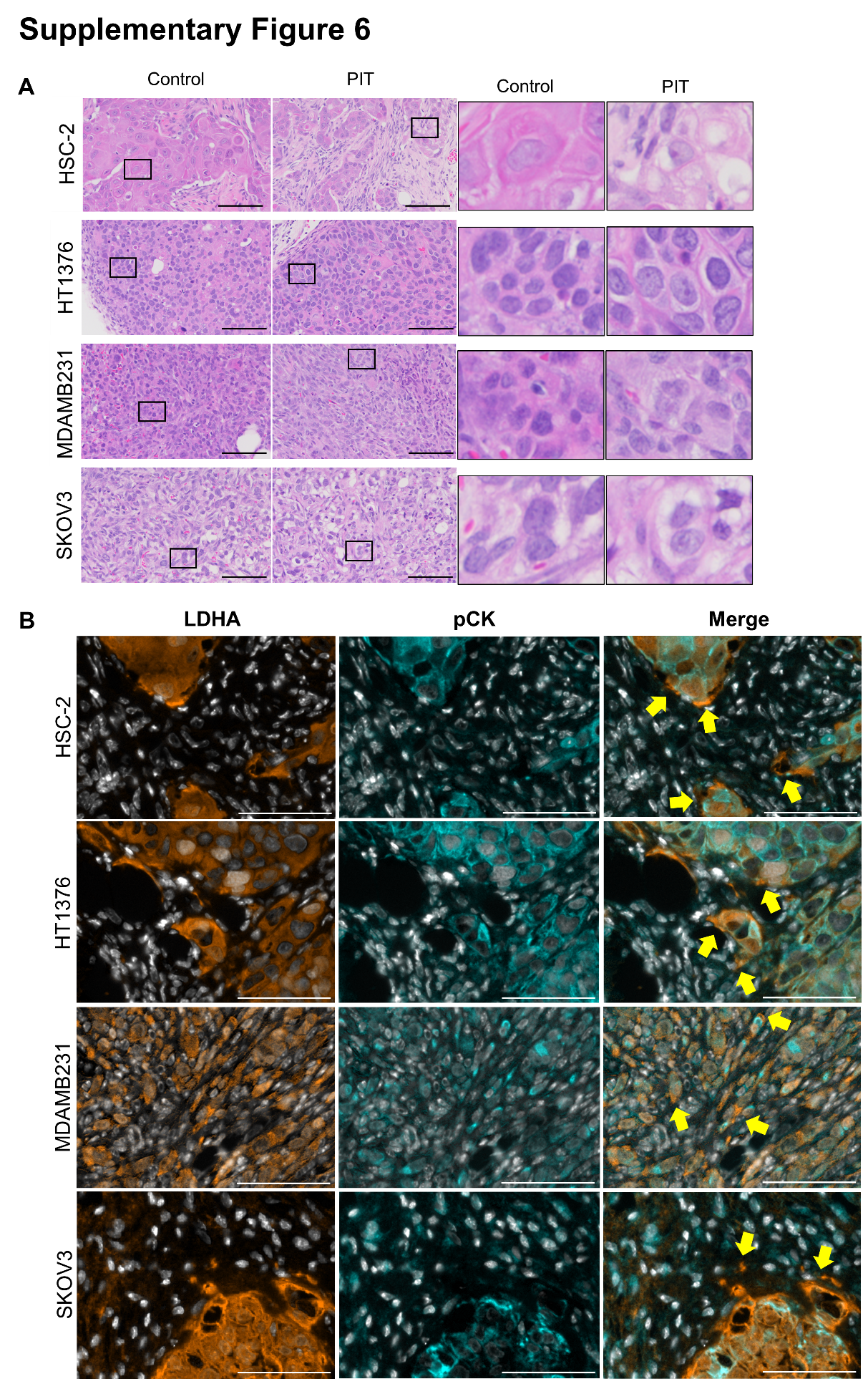


**Supplementary Fig. S6. Histological changes after in vivo TF-targeted NIR-PIT.**

(A) H&E staining of HSC-2, HT1376-luc, MDAMB231, and SKOV3-luc tumors after NIR-PIT (images, × 200; scale bar, 100 μm). Insets are enlarged and displayed in the right panels. (B) Immunohistochemical evaluation of LDHA expression in HSC-2, HT1376-luc, MDAMB231, and SKOV3-luc tumors 24 hours after NIR-PIT. Representative micrographs of LDHA expression (images; ×200; scale bar, 100 μm). The inset shows examples of LDHA leakage into the extracellular space, which suggests necrotic cell death (yellow-filled arrow). Antibody staining of LDHA and pan-cytokeratin (pCK) is shown in orange and cyan, respectively. Nuclei are stained with DAPI and shown in white.
